# Long term impact of large scale community-directed delivery of doxycycline for the treatment of onchocerciasis

**DOI:** 10.1186/1756-3305-5-53

**Published:** 2012-03-20

**Authors:** Francesca Tamarozzi, Nicholas Tendongfor, Peter A Enyong, Mathias Esum, Brian Faragher, Samuel Wanji, Mark J Taylor

**Affiliations:** 1 Molecular and Biochemical Parasitology, Liverpool School of Tropical Medicine, Pembroke Place, Liverpool L3 5QA, UK; 2 Department of Microbiology and Parasitology, University of Buea, P.O. Box 63, Buea, Cameroon; 3 Research Foundation for Tropical Diseases and Environment, P.O. Box 474, Buea, Cameroon; 4 Tropical Medicine Research Station, P.O. Box 55, Kumba, Cameroon; 5 Clinical Group, Liverpool School of Tropical Medicine, Pembroke Place, Liverpool L3 5QA, UK

**Keywords:** *Onchocerca volvulus*, *Wolbachia*, Doxycycline, Community-directed delivery, Effectiveness

## Abstract

**Background:**

Anti-*Wolbachia *treatment with doxycycline is effective in sterilising and killing adult *Onchocerca volvulus *nematodes, proving superior to ivermectin and of great potential as an alternative approach for the treatment and control of onchocerciasis, particularly in areas of *Loa loa *co-endemicity. Nevertheless, the length of the required treatment poses potential logistical problems and risk of poor compliance, raising a barrier to the use of doxycycline in Mass Drug Administration (MDA) strategies. In 2007 and 2008 a feasibility trial of community-directed treatment with doxycycline was carried out in two health districts in Cameroon, co-endemic for *O. volvulus *and *L. loa*. With 17,519 eligible subjects, the therapeutic coverage was 73.8% with 97.5% compliance, encouraging the feasibility of using doxycycline community-directed delivery in restricted populations of this size. The current study evaluated the effectiveness of this community-directed delivery of doxycycline four years after delivery.

**Findings:**

Infection with *O. volvulus *was evaluated by skin biopsy and nodule palpation. Of the 507 subjects recruited, 375 had completed the treatment with doxycycline followed by one or two rounds of annual ivermectin MDA and 132 received one or two rounds of annual ivermectin MDA alone. Statistically significant lower microfilarial prevalence (17.0% [doxycycline plus ivermectin group], 27.0% [ivermectin only group], *p *= 0.014) and load (*p *= 0.012) were found in people that had received doxycycline followed by ivermectin compared to those who received ivermectin only.

**Conclusions:**

This study demonstrates the long-term effectiveness of doxycycline treatment delivered with a community-directed strategy even when evaluated four years after delivery in an area of ongoing transmission. This finding shows that a multi-week course of treatment is not a barrier to community-delivery of MDA in restricted populations of this size and supports its implementation to compliment existing control strategies for onchocerciasis, where needed.

## Background

Onchocerciasis or 'river blindness' is a debilitating disease affecting an estimated 37 million people in Sub Saharan Africa and Latin America, with at least 500,000 people visually impaired and 270,000 blinded by the disease [1]. Annual or biannual Mass Drug Administration (MDA) of ivermectin is the current strategy implemented by the African Programme for Onchocerciasis Control [2]. At the standard dose of 150 μg/kg, it is highly effective at reducing dermal microfilarial loads, but is only marginally active against adult worms [3,4], requiring sustained delivery for at least 15-17 years in order to interrupt transmission in areas of moderate transmission [5]. Moreover, the severe and occasionally fatal adverse reactions following ivermectin intake in people with high loads of *Loa loa *microfilariae (mf) are an obstacle to the implementation of onchocerciasis control programmes in areas of co-endemicity [6,7].

Four to six week courses of doxycycline have been proven in clinical trials to be highly effective at blocking embryogenesis of adult worms, and leading to a macrofilaricidal rate of 60-70% [8-10]. This demonstrates superior efficacy of doxycycline compared to ivermectin, and provides an important alternative treatment for onchocerciasis in areas of *L. loa *co-endemicity, in areas where evidence of sub-optimal efficacy of ivermectin occurs [11-13], and in programme endgame situations [14] to achieve elimination of the infection. Doxycycline targets the bacterial endosymbiont *Wolbachia*, and so can be used safely in areas where the *Wolbachia*-free *L. loa *is co-endemic, as it has no microfilaricidal activity against *L. loa *[10].

By virtue of its pronounced macrofilaricidal and embryostatic effects, it is expected that community-wide distribution of doxycycline will lead to long-term reductions in microfilaridermia. Nevertheless, one barrier to the widespread use of doxycycline is the perceived risk of poor compliance due to the length of treatment regimes [15]. In a phase III trial, Wanji and colleagues countered this perception by demonstrating the feasibility and safety of large-scale delivery of doxycycline using a community-directed strategy in areas of *L. loa *co-endemicity in Cameroon, naive to onchocerciasis control programmes [16]. Of 17,519 eligible subjects, 12,936 were treated with doxycycline in 2007 or 2008, giving a therapeutic coverage of 73.83% for the eligible population. Moreover, the 6 week course of treatment was completed by 97.5% of individuals who started it, as assessed by directly observed drug intake. Following community-directed delivery of doxycycline, communities received two rounds of annual ivermectin MDA, which was distributed as part of the national control programme that was extended to these areas. In addition to showing a high level of coverage and compliance to a 6 week course of doxycycline, the feasibility study highlighted the safety of this regime, in which there were no Severe Adverse Events (SAEs) in any of the 17,519 people during treatment with doxycycline, yet when treated with ivermectin two SAEs were reported, as observed in other *L. loa *co-endemic areas [6,7]. The current single-blind evaluation trial was carried out to assess the long-term effectiveness of this community-directed treatment with doxycycline in reducing skin microfilarial prevalence and load four years after implementation in an area of ongoing infection transmission.

## Methods

The study was approved by the Research Ethics Committee of the Liverpool School of Tropical Medicine (UK) and the Institutional Review Board of the Medical Research Station of Kumba (Cameroon), and registered in the ISRCTN register (ISRCTN95189962). The study was carried out in three health areas of Mbanga health district in the littoral region of Cameroon. Community-directed delivery of doxycycline was implemented in 2007 and 2008 [16], followed by two rounds of ivermectin MDA, as part of the national control programme. Participants of both sexes, aged 19 or above, were recruited from those who completed the 6 week course of doxycycline MDA in 2007 or 2008, plus one or two annual treatments of ivermectin MDA, and those who received ivermectin MDA alone. End-points were microfilarial prevalence (primary outcome) and load in the two treatment groups. Presence and number of palpable nodules was also assessed.

For the sample size calculation, the expected reduction in microfilaridermia prevalence in the two groups was estimated using the Rapid Evidence Assessment of *O. volvulus *prevalence in the investigated areas [16,17] and its expected change after the implemented treatments [10,12]. A sample size of 500 subjects had a 90% power to detect a difference in prevalence of microfilaridermia of 16.0% (expected prevalence in the doxycycline plus ivermectin group) versus 26.8%. This was considered the smallest difference between groups relevant for effectiveness assessment. Moreover, this sample size was considered adequate to obtain an accurate estimate of differences between groups and also in the light of the expected microfilaridermia prevalence after two rounds of ivermectin MDA (58.6%).

Villages in each health area were randomly selected and inhabitants were invited to participate in the study. After a thorough explanation of the study's aim and execution in the local language, all participants gave informed consent prior to examination. For the assessment of microfilarial prevalence and load, two skin biopsies (skin snips) from the iliac crests were taken using a sterile corneoscleral punch and incubated in saline solution in microtitre plates overnight (10-18 h). The number of mf was counted and the skin biopsies weighed to calculate the number of mf per mg of skin (mf/mg). Body palpation was carried out to assess the presence and number of palpable onchocercal nodules. Both mf counting and body palpation were carried out in a blinded manner with regard to the treatment group.

### Statistical analysis

Differences in microfilarial and nodule prevalence between groups were assessed by Fisher's Exact test. Differences in microfilarial and nodule burden between groups were assessed by Mann-Whitney *U *test. A p-value ≤ 0.05 was considered significant. All analysis was carried out using SPSS Statistics 17.0 (IBM).

## Results

Five-hundred and seven people, 260 males and 247 females, were recruited from 17 villages of the three health areas investigated. Of those, 375 had completed the treatment with doxycycline followed by one or two annual rounds of ivermectin MDA and 132 received one or two annual rounds of ivermectin MDA alone (Table 1). There was no difference in the proportion of people who had received one versus two rounds of ivermectin between the groups (53.1% [one round of ivermectin in doxycycline plus ivermectin group], 59.8% [one round of ivermectin in ivermectin only group], *p *= 0.858).

**Table 1:** Characteristics of the investigated population

Treatment group	Health district	Males (%)	Females (%)	Total	Mean age years (range)
**Doxycycline + ivermectin**	**All**	**210 (56%)**	**165 (44%)**	**375**	**43.16 (19-81)**
	
	**Matouke**	104 (57.14%)	78 (42.86%)	182	43.66 (19-74)
	
	**Mombo**	68 (54.84%)	56 (45.16%)	124	42.39 (19-81)
	
	**Kotto**	38 (55.07%)	31 (44.93%)	69	43.23 (23-78)

**Ivermectin only**	**All**	**50 (37.88%)**	**82 (62.12%)**	**132**	**36.86 (19-71)**
	
	**Matouke**	17 (26.56%)	47 (73.44%)	64	34.78 (19-62)
	
	**Mombo**	14 (53.85%)	12 (46.15%)	26	40.54 (20-69)
	
	**Kotto**	19 (45.24%)	23 (54.76%)	42	38.00 (20-71)

**Figure 1:**
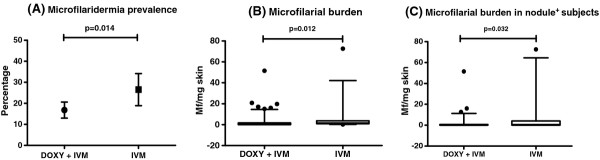
**Differences in microfilarial prevalence and load between groups**. Investigated groups are people who received community-directed doxycycline followed by one or two rounds of annual ivermectin MDA (DOXY + IVM, N = 375) and those who received one or two rounds of annual ivermectin MDA only (IVM, N = 132). A) Difference in microfilaridermia prevalence between groups; the graph shows mean and 95% CI. Fisher's Exact test. B) Difference in microfilarial loads between groups; the graph shows median and 5-95 percentiles of mf/mg of skin in Mf^+ ^subjects. Mann-Whitney *U *test. C) Difference in microfilarial loads in people with palpable nodules (DOXY+IVM, N = 70; IVM, N = 26); the graph shows median and 5-95 percentiles of mf/mg of skin. Mann-Whitney *U *test.

Microfilaridermia prevalence was significantly lower in people who received doxycycline followed by one or two annual rounds of ivermectin MDA compared to those who received ivermectin MDA alone (*p *= 0.014, Figure 1A and Table 2). The percentage of amicrofilaridermic people was 83.2% in the group that had received doxycycline followed by ivermectin MDA compared to 73.2% in the group that had received only ivermectin MDA. A significant difference was also found in microfilarial burden (*p *= 0.012, Figure 1B and Table 2), with people that had received doxycycline harbouring significantly lower microfilarial loads. When evaluating those with palpable nodules, we found that microfilarial loads were significantly lower in those who received doxycycline (*p *= 0.032, Figure 1C and Table 2). In this subset of people, microfilaridermia prevalence was also lower in the group that had received doxycycline, even though it did not reach statistical significance (*p *= 0.091, Table 2). No differences were detected in palpable nodule prevalence and burden between the groups.

**Table 2:** Parasitological results and statistical differences between groups four years after doxycycline MDA implementation

	Treatment group		
	
	Doxycycline + ivermectin	Ivermectin only	p-value
**Mf prevalence (%)** **(95% CI)**	17% (13%-21%)	27% (19%-35%)	**0.014**

**Mf/mg skin** **Median (75-95 percentile)**	0 (0-3.5)	0 (0.37-5.37)	**0.012**

**Nodule prevalence (%)** **(95% CI)**	19% (15%-23%)	20% (13%-27%)	0.776

**Nodules/person** **Median (75-95 percentile)**	0 (0-2)	0 (0-2)	0.720

**Mf prevalence in nodule^+^subjects (%)** **(95% CI)**	34% (23%-46%)	54% (33%-74%)	0.091

**Mf/mg skin in nodule^+^subjects** **Median (75-95 percentile)**	0 (0.71-11.33)	0 (4.00-64.53)	**0.032**

## Discussion

Our findings show that delivery of doxycycline with a community-directed approach is not only feasible, but also effective in reducing microfilaridermia prevalence and loads. Importantly, these results were obtained four years after implementation in an area of ongoing transmission and naive to previous control measures, and so demonstrate the long-term efficacy of community-wide doxycycline treatment.

Although this study demonstrates the feasibility and effectiveness of doxycycline treatment, the contraindications of this drug in children < 9 years old and in pregnancy and the desirability for shorter treatment regimes has driven the formation of the A·WOL consortium, which aims to discover and develop alternative anti-wolbachial drugs and regimes that overcome exclusion of these patient groups and reduce treatment timeframes [18].

The lack of difference in nodule prevalence and burden between groups most likely reflects the ongoing transmission of the infection in the area, where new incoming parasites may colonise already existing nodules [19]. Also, although doxycycline has been demonstrated in phase II trials to show a high rate of macrofilaricidal activity (60-70%) [8,10,19], the rate of nodule re-absorption is not known. All these factors, therefore, make the use of palpable nodule prevalence and burden less suitable to assess the effectiveness of doxycycline in an area of ongoing transmission and within the investigated timeframe from distribution.

Our results should encourage the introduction of community-directed distribution of doxycycline and other anti-wolbachial drugs and regimes in onchocerciasis control programmes, such as in restricted populations with high risk of SAEs to ivermectin in *L. loa *co-endemic areas, in populations with evidence of sub-optimal responsiveness to ivermectin and as an endgame tool to meet the goal of elimination (as planned for the North-East focus of onchocerciasis in Venezuela as part of the Onchocerciasis Elimination Programme for the Americas [OEPA]). Moreover, as doxycycline is the first macrofilaricidal drug available for onchocerciasis, knowledge of its long-term impact is of particular importance to address the practical aspects of implementation of macrofilaricides within existing control programmes for onchocerciasis.

## Abbreviations

MDA: Mass drug administration; Mf: Microfilariae; SAEs: Severe adverse events

## Competing interests

The authors declare that they have no competing interests.

## Authors' contributions

FT designed the study, participated in the field activity, analysed and interpreted the results and wrote the manuscript. NT participated in the field activity organization and data collection. PAE participated in the field activity organization and body palpation. ME participated in the field activity organization and data collection. BF participated in the study design and statistical analysis of data. SWJ participated in the study design, organized the field activity and participated in the interpretation of the data and manuscript editing. MJT conceived and designed the study, interpreted the results and wrote the manuscript. All authors agreed with the final edition of the manuscript.
